# Beyond species means – the intraspecific contribution to global wood density variation

**DOI:** 10.1111/nph.70860

**Published:** 2026-01-16

**Authors:** Fabian Jörg Fischer, Jérôme Chave, Amy Zanne, Tommaso Jucker, Alex Fajardo, Adeline Fayolle, Renato Augusto Ferreira de Lima, Ghislain Vieilledent, Hans Beeckman, Wannes Hubau, Tom De Mil, Daniel Wallenus, Ana María Aldana, Esteban Alvarez‐Dávila, Luciana F. Alves, Deborah M. G. Apgaua, Fátima Arcanjo, Jean‐François Bastin, Andrii Bilous, Philippe Birnbaum, Volodymyr Blyshchyk, Joli Borah, Vanessa Boukili, J. Julio Camarero, Luisa Casas, Roberto Cazzolla Gatti, Jeffrey Q. Chambers, Ezequiel Chimbioputo Fabiano, Brendan Choat, Georgina Conti, Will Cornwell, Javid Ahmad Dar, Ashesh Kumar Das, Magnus Dobler, Dao Dougabka, David P. Edwards, Robert Evans, Daniel Falster, Philip Fearnside, Olivier Flores, Nikolaos Fyllas, Jean Gérard, Rosa C. Goodman, Daniel Guibal, L. Francisco Henao‐Diaz, Vincent Hervé, Peter Hietz, Jürgen Homeier, Thomas Ibanez, Jugo Ilic, Steven Jansen, Rinku Moni Kalita, Tanaka Kenzo, Liana Kindermann, Subashree Kothandaraman, Martyna Kotowska, Yasuhiro Kubota, Patrick Langbour, James Lawson, André Luiz Alves de Lima, Roman Mathias Link, Anja Linstädter, Rosana López, Cate Macinnis‐Ng, Luiz Fernando S. Magnago, Adam R. Martin, Ashley M. Matheny, James K. McCarthy, Regis B. Miller, Arun Jyoti Nath, Bruce Walker Nelson, Marco Njana, Euler Melo Nogueira, Alexandre Oliveira, Rafael Oliveira, Mark Olson, Yusuke Onoda, Keryn Paul, Daniel Piotto, Phil Radtke, Onja Razafindratsima, Tahiana Ramananantoandro, Jennifer Read, Sarah Richardson, Enrique G. de la Riva, Oris Rodríguez‐Reyes, Samir G. Rolim, Victor Rolo, Julieta A. Rosell, Roberto Salguero‐Gómez, Nadia S. Santini, Bernhard Schuldt, Luitgard Schwendenmann, Arne Sellin, Timothy Staples, Pablo R. Stevenson, Somaiah Sundarapandian, Masha T. van der Sande, Bernard Thibaut, David Yue Phin Tng, José Marcelo Domingues Torezan, Boris Villanueva, Aaron Weiskittel, Jessie Wells, S. Joseph Wright, Kasia Zieminska

**Affiliations:** ^1^ School of Biological Sciences University of Bristol Bristol BS8 1TQ UK; ^2^ Centre de Recherche Biodiversité Environnement UMR 5300 (CNRS/IRD/UPS/INPT) Toulouse Cedex 9 31062 France; ^3^ School of Life Sciences, Ecosystem Dynamics and Forest Management in Mountain Landscapes Technical University of Munich Hans‐Carl‐von‐Carlowitz‐Platz 2 Freising 85354 Germany; ^4^ Cary Institute of Ecosystem Studies Millbrook NY 12545 USA; ^5^ Dirección de Investigación, Vicerrectoría Académica Universidad de Talca Campus Lircay Talca 3460000 Chile; ^6^ Instituto de Ecología y Biodiversidad (IEB) Las Palmeras 3425 Ñuñoa 8310000 Chile; ^7^ Millenium Nucleus of Patagonian Limit‐of‐Life (LiLi) Valdivia 5090000 Chile; ^8^ TERRA Teaching and Research Centre Gembloux Agro Bio‐Tech, Université de Liège Gembloux B‐5030 Belgium; ^9^ CIRAD UPR Forêts et Sociétés Montpellier F‐34398 France; ^10^ Departamento de Ciências Biológicas, ESALQ Universidade de São Paulo Avenida Pádua Dias, 11 Piracicaba 13418‐900 Brazil; ^11^ AMAP Université de Montpellier, CIRAD, CNRS, INRAE, IRD Montpellier F‐34398 France; ^12^ CIRAD UMR AMAP Nouméa Nouvelle‐Calédonie F‐98848 France; ^13^ IAC Nouméa Nouvelle‐Calédonie F‐98848 France; ^14^ Service of Wood Biology Royal Museum for Central Africa Tervuren B‐3080 Belgium; ^15^ Department of Forest and Water Management, Laboratory of Wood Technology Ghent University Ghent B‐9000 Belgium; ^16^ Laboratorio de Ecología de Bosques Tropicales y Primatología Universidad de Los Andes Bogotá DC 111711 Colombia; ^17^ Fundación Con Vida Calle 9 # 43A – 33, Oficina 306, Multicentro Aliadas Medellín Colombia; ^18^ Center for Tropical Research Institute of the Environment and Sustainability, UCLA Los Angeles CA 90095‐1496 USA; ^19^ Centre for Rainforest Studies The School for Field Studies Yungaburra Qld 4872 Australia; ^20^ Universidade Federal de Uberlândia Uberlândia 38408‐100 Brazil; ^21^ Biodiversity and Ecosystem Restoration Lab Londrina State University Campus Universitario, CCB, BAV Londrina PR 86051‐990 Brazil; ^22^ National University of Life and Environmental Sciences of Ukraine (NUBiP) Kyiv 03041 Ukraine; ^23^ Hochschule Weihenstephan‐Triesdorf Freising 85354 Germany; ^24^ CIRAD, UMR AMAP Montpellier F‐34398 France; ^25^ Department of Animal and Plant Sciences University of Sheffield Sheffield S1 4DE UK; ^26^ Department of Ecology and Evolutionary Biology University of Connecticut Storrs CT 06269 USA; ^27^ Office of Strategic Planning and Community Development Somerville MA 02143 USA; ^28^ Instituto Pirenaico de Ecología (IPE‐CSIC) Avda. Montañana Zaragoza 1005 50192 Spain; ^29^ Laboratorio de Ecología de Bosques Tropicales y Primatología Universidad de Los Andes Bogotá D.C. 111711 Colombia; ^30^ Instituto de Investigación de Recursos Biológicos Alexander von Humboldt Cl 72 #12 ‐ 65 Piso 4 Bogotá Colombia; ^31^ Department of Biological, Geological and Environmental Sciences University of Bologna Bologna 40126 Italy; ^32^ Department of Geography University of California, Berkeley Berkeley CA 94709 USA; ^33^ Department of Wildlife Management and Tourism Studies University of Namibia Katima‐Mulilo Campus Ngweze Katima‐Mulilo 1096 Namibia; ^34^ Hawkesbury Institute for the Environment Western Sydney University Richmond NSW 2753 Australia; ^35^ Instituto Multidisciplinario de Biología Vegetal (IMBIV) CONICET ‐ Universidad Nacional de Córdoba, Edificio de Investigaciones Científicas y Técnicas Av. Vélez Sársfield 1611 Córdoba CP5000 Argentina; ^36^ School of Biological, Earth and Environmental Sciences University of New South Wales Sydney NSW 2033 Australia; ^37^ Terrestrial Ecology and Modelling (TEaM) Lab, Department of Environmental Science and Engineering SRM University‐AP Amaravati Andhra Pradesh 522240 India; ^38^ Centre for Geospatial Technology SRM University‐AP Amaravati Andhra Pradesh 522240 India; ^39^ Department of Ecology and Environmental Science Assam University Silchar Assam 788 011 India; ^40^ Biodiversity Research and Systematic Botany, Institute of Biochemistry and Biology, Faculty of Science University of Potsdam Potsdam 14469 Germany; ^41^ Département des Sciences Fondamentales École Nationale Supérieure des Travaux Publics du Tchad N'Djamena BP 60 Chad; ^42^ Department of Plant Sciences and Centre for Global Wood Security University of Cambridge Cambridge CB2 3EA UK; ^43^ Conservation Research Institute University of Cambridge Cambridge CB2 3EA UK; ^44^ School of Agriculture, Food and Ecosystem Sciences University of Melbourne Burnley Campus, Boulevard Drive Richmond VIC 3121 Australia; ^45^ Evolution and Ecology Research Centre University of New South Wales Sydney NSW 2052 Australia; ^46^ National Institute for Research in Amazonia (INPA) Manaus Amazonas CEP 69067‐375 Brazil; ^47^ Université de La Réunion UMR PVBMT Saint‐Pierre Reunion 97410 France; ^48^ Department of Biology Section of Ecology and Taxonomy Athens GR‐15772 Greece; ^49^ CIRAD UPR BioWooEB Montpellier F‐34398 France; ^50^ BioWooEB Univ Montpellier, Cirad Montpellier F‐34398 France; ^51^ Department of Forest Ecology and Management Swedish University of Agricultural Sciences (SLU) Umeå 901 83 Sweden; ^52^ Division of Physical Resource Theory, Department of Space, Earth and Environment Chalmers University of Technology Gothenburg 412 96 Sweden; ^53^ University and Jepson Herbaria University of California Berkeley CA 94720 USA; ^54^ INRAE, AgroParisTech, UMR SayFood Université Paris‐Saclay Palaiseau 91120 France; ^55^ Institute of Botany, Department of Ecosystem Management, Climate and Biodiversity BOKU University Vienna 1180 Austria; ^56^ Faculty of Resource Management HAWK University of Applied Sciences and Arts Daimlerstraße 2 Göttingen 37075 Germany; ^57^ Plant Ecology and Ecosystems Research Georg‐August University of Göttingen Göttingen 37073 Germany; ^58^ School of Biological, Earth and Environmental Sciences, Evolution & Ecology Research Centre, Faculty of Science University of New South Wales Kennington NSW 2033 Australia; ^59^ Department of Forest and Ecosystem Science The University of Melbourne Melbourne VIC 3010 Australia; ^60^ Institute of Botany Ulm University Albert‐Einstein‐Allee 11 Ulm 89081 Germany; ^61^ Department of Botany Bhattadev University Bajali Assam 781325 India; ^62^ Japan International Research Center for Agricultural Sciences Tsukuba Ibaraki 305‐8686 Japan; ^63^ Terrestrial Ecology and Modelling (TEaM) Lab, Department of Environmental Science and Engineering SRM University‐AP Amaravati Andhra Pradesh 522502 India; ^64^ Centre for Geospatial Technology SRM University‐AP Amaravati Andhra Pradesh 522502 India; ^65^ School of Natural Sciences Macquarie University Sydney NSW 2109 Australia; ^66^ Department of Plant Ecology and Ecosystems Research, Albrecht‐von‐Haller Institute for Plant Sciences University of Goettingen Göttingen 37077 Germany; ^67^ Think Nature Inc. Urasoe City Okinawa 901‐2102 Japan; ^68^ Faculty of Science University of the Ryukyus Nishihara Okinawa 903‐0129 Japan; ^69^ NSW Department of Primary Industries and Regional Development Climate and Natural Resources Group Ourimbah NSW 2250 Australia; ^70^ Serra Talhada Academic Unit Federal Rural University of Pernambuco Serra Talhada PE CEP: 56909‐535 Brazil; ^71^ Forest Botany Technical University of Dresden Pienner Straße 7 Tharandt 01737 Germany; ^72^ Departamento de Sistemas y Recursos Naturales, Escuela Técnica Superior de Ingenieros de Montes Universidad Politécnica de Madrid Madrid 28040 Spain; ^73^ School of Biological Sciences and Te Pūnaha Matatini, Waipapa Taumata Rau University of Auckland Auckland 1010 New Zealand; ^74^ Universidade Federal do Sul da Bahia Rodovia Ilhéus/Itabuna, Km 22, CEPLAC‐CEPEC Ilhéus BA 45604‐811 Brazil; ^75^ Department of Physical and Environmental Sciences University of Toronto Scarborough Toronto ON M1C 1A4 Canada; ^76^ Jackson School of Geosciences, Department of Earth and Planetary Sciences University of Texas at Austin Austin TX 78712‐1692 USA; ^77^ Manaaki Whenua – Landcare Research Lincoln 7608 New Zealand; ^78^ Self‐employed; ^79^ Instituto Nacional de Pesquisas da Amazônia Manaus AM CEP 69067‐375 Brazil; ^80^ Wildlife Conservation Society Tanzania Country Program, Nature‐Based Solutions P. O Box 5196 Dar es Salaam Tanzania; ^81^ Centro Universitário UniFG Av. Governador Nilo Coelho, 4911, São Sebastião Guanambi Bahia CEP 46430‐000 Brazil; ^82^ Colégio Estadual Francisco Moreira Alves Jaborandi Bahia 47655‐000 Brazil; ^83^ Instituto de Biociências Cidade Universitária, Universidade de São Paulo São Paulo SP CEP: 05508‐090 Brazil; ^84^ Departamento de Biologia Vegetal Instituto de Biologia, Centro de Ecologia Integrativa, Universidade Estadual de Campinas Campinas CEP 13083‐862 Brazil; ^85^ Departamento de Botánica, Instituto de Biología Universidad Nacional Autónoma de México Ciudad de México C.P. 04510 Mexico; ^86^ Division of Forest and Biomaterials Sciences, Graduate School of Agriculture Kyoto University Kyoto 606‐8502 Japan; ^87^ CSIRO Environment Canberra ACT ACT 2601 Australia; ^88^ Centro de Formação em Ciências Agroflorestais Universidade Federal do Sul da Bahia BR 415, km 29 Ilhéus BA 45613‐204 Brazil; ^89^ Department of Forest Resources & Environmental Conservation Virginia Tech 319E Cheatham Hall Blacksburg VA 24061 USA; ^90^ University of California Berkeley Berkeley CA 94720 USA; ^91^ Mention Foresterie et Environnement University of Antananarivo, Ecole Supérieure des Sciences Agronomiques BP 175 Antananarivo 101 Madagascar; ^92^ School of Biological Sciences Monash University Melbourne VIC 3800 Australia; ^93^ Area de Ecología, Facultad de Ciencias Biológicas y Ambientales, Departamento de Biodiversidad y Gestión Ambiental Universidad de León Campus de Vegazana s/n León 24071 Spain; ^94^ Smithsonian Tropical Research Institute Ancón Panama City Panama; ^95^ Instituto de Ciencias Ambientales y Biodiversidad Universidad de Panamá, Estafeta Universitaria Panama City Panama; ^96^ Forest Research Group, INDEHESA University of Extremadura Plasencia 10600 Spain; ^97^ Laboratorio Nacional de Ciencias de la Sostenibilidad, Instituto de Ecología, Universidad Nacional Autónoma de México Tercer Circuito sn de Ciudad Universitaria Mexico City 04510 Mexico; ^98^ Department of Biology University of Oxford Oxford OX1 3EL UK; ^99^ Pembroke College University of Oxford Oxford OX1 1DW UK; ^100^ Instituto de Geología Universidad Nacional Autónoma de México, Circuito de la Investigación Científica, Ciudad Universitaria Ciudad de México C.P. 04510 México; ^101^ Laboratorio Nacional de Geoquímica y Mineralogía Universidad Nacional Autónoma de México, Circuito de la Investigación Científica, Ciudad Universitaria Ciudad de México C.P. 04510 México; ^102^ Plant Ecology, Albrecht von Haller Institute for Plant Sciences University of Goettingen Untere Karspüle 2 Göttingen D‐37073 Germany; ^103^ School of Environment University of Auckland Auckland 1010 New Zealand; ^104^ Institute of Ecology and Earth Sciences University of Tarty Tartu 50409 Estonia; ^105^ School of the Environment The University of Queensland St Lucia Qld 4067 Australia; ^106^ Department of Ecology and Environmental Sciences Pondicherry University Puducherry 605014 India; ^107^ Forest Ecology and Forest Management Group Wageningen University & Research Wageningen 6708 the Netherlands; ^108^ LMGC Univ Montpellier, CNRS Montpellier F‐34090 France; ^109^ Universidad del Tolima GIBDET Ibagué Tolima 730006299 Colombia; ^110^ Jardín Botánico de Bogotá Av. Calle 63 # 68‐95 Bogotá Colombia; ^111^ Center for Research on Sustainable Forests University of Maine Orono ME 04469 USA; ^112^ School of Geography, Earth and Atmospheric Sciences The University of Melbourne Carlton VIC 3053 Australia; ^113^ Smithsonian Tropical Research Institute Balboa Panama; ^114^ Independent Researcher

**Keywords:** aridity, biomass, functional trait, hierarchical modelling, intraspecific variation, wood density

## Abstract

Wood density is central for estimating vegetation carbon storage and a plant functional trait of great ecological and evolutionary importance. However, the global extent of wood density variation is unclear, especially at the intraspecific level. We assembled the most comprehensive wood density collection to date, including 109 626 records from 16 829 plant species across woody life forms and biomes (GWDD v.2, available here: doi: 10.5281/zenodo.16919509). Using the GWDD v.2, we explored the sources of wood density variation within individuals, within species and across environmental gradients. Intraspecific variation accounted for *c*. 15% of overall wood density variation (SD = 0.068 g cm^−3^). Variance was 50% smaller in sapwood than heartwood, and 30% smaller in branchwood than trunkwood. Individuals in extreme environments (dry, hot and acidic soils) had higher wood density than conspecifics elsewhere (+0.02 g cm^−3^, *c*. 4% of the mean). Intraspecific environmental effects strongly tracked interspecific patterns (*r* = 0.83) but were 70–80% smaller and varied considerably among taxa. Individual plant wood density was difficult to predict (root mean square error > 0.08 g cm^−3^; single‐measurement *R* ^2^ = 0.59). We recommend: (1) systematic sampling of multiple individuals and tissues for local applications; and (2) expanded taxonomic coverage combined with integrative models for robust estimates across ecological scales.

## Résumé

La densité du bois est essentielle pour estimer le stockage de carbone de la végétation et constitue un trait fonctionnel d’une grande importance écologique et évolutive. Cependant, l’ampleur de la variation globale de la densité du bois reste mal caractérisée, en particulier au niveau intraspécifique. Nous avons rassemblé la base de données la plus complète à ce jour sur la densité du bois, comprenant 109 626 mesures provenant de 16 829 espèces de plantes ligneuses appartenant à diverses formes de vie et biomes (GWDD v.2, disponible ici: doi: 10.5281/zenodo.16919509). À l’aide de la GWDD v.2, nous avons exploré les sources de variation de la densité du bois au sein des individus, au sein des espèces et le long de gradients environnementaux. La variation intraspécifique représentait environ 15% de la variation totale de la densité du bois (SD = 0,068 g cm^−3^). La variance était 50% plus faible dans l’aubier que dans le duramen, et 30% plus faible dans le bois des branches que dans celui du tronc. Les individus vivant dans des environnements extrêmes (secs, chauds, sols acides) présentaient une densité du bois plus élevée que leurs congénères ailleurs (+0,02 g cm^−3^, soit ~4% de la moyenne). Les effets environnementaux intraspécifiques reflétaient les tendances interspécifiques (*r* = 0,83), mais étaient 70 à 80% plus faibles et variaient considérablement entre taxons. La densité du bois à l’échelle individuelle était difficile à prédire (RMSE >0,08 g cm^−3^; *R* ^2^ = 0,59). Nous recommandons (1) un échantillonnage systématique de plusieurs individus et tissus pour les applications locales et (2) un élargissement de la couverture taxonomique combiné à des modèles intégratifs afin d’obtenir des estimations robustes à travers les échelles écologiques.

## Resumen

La densidad de la madera es fundamental para estimar el almacenamiento de carbono vegetal y constituye un rasgo funcional de gran importancia ecológica y evolutiva. Sin embargo, el alcance global de la variación de la densidad de madera no está claramente descrito, especialmente a nivel intraespecífico. Recopilamos la colección más completa de densidad de madera hasta la fecha, que incluye 109 626 registros de 16 829 especies de plantas leñosas de diversas formas de vida y biomas (GWDD v.2, disponible aquí: doi: 10.5281/zenodo.16919509). Utilizando la GWDD v.2, exploramos las fuentes de variación en la densidad de la madera entre individuos y especies, así como a través de gradientes ambientales. La variación intraespecífica representó aproximadamente el 15% de la variación total de la densidad de la madera (SD = 0.068 g cm^−3^). La varianza fue un 50% menor en la albura que en el duramen, y un 30% menor en la madera de ramas que en la del tronco. Los individuos en ambientes extremos (secos, cálidos, suelos ácidos) presentaron una densidad de madera mayor que sus congéneres en otros lugares (+0.02 g cm^−3^, ~4% de la media). Los efectos ambientales intraespecíficos siguieron estrechamente los patrones interespecíficos (*r* = 0.83), sin embargo, resultaron entre un 70% y un 80% menores y variaron considerablemente entre los taxones. La densidad de madera por individuo fue difícil de predecir (RMSE >0.08 g cm^−3^; *R* ^2^ de medición única = 0.59). Recomendamos (1) el muestreo sistemático intraespecífico de múltiples individuos para aplicaciones locales y (2) una cobertura taxonómica ampliada combinada con modelos integradores para obtener estimaciones robustas a través de las escalas ecológicas.

1

**Table:** 

Content		
	Summary	2633
I.	Introduction	2633
II.	Materials and Methods	2635
III.	Results	2638
IV.	Discussion	2641
	Acknowledgements	2646
	References	2647

## I. Introduction

Wood density, the oven‐dry mass of wood (g) over its fresh volume (cm^−3^), is an important plant functional trait in ecology and global change studies. Accurate species‐level averages of wood density are needed for unbiased estimation of aboveground carbon in vegetation (Phillips *et al*., 2019). Moreover, wood density defines one of the main axes of global plant trait variation (Díaz *et al*., 2016). Generally, high‐wood density species are less susceptible to mechanical, hydraulic or biotic stress (Chave *et al*., 2009), experience low mortality at the expense of growth (King *et al*., 2006; Kraft *et al*., 2010) and decompose more slowly (Hérault *et al*., 2010). Wood density therefore displays distinct patterns across successional and environmental gradients (Šímová *et al*., 2018; Poorter *et al*., 2019) and is a key factor in the prediction of the global carbon cycle and terrestrial ecosystem dynamics (Sakschewski *et al*., 2015).

Wood density varies widely among woody plants, from species with incredibly low‐density wood (*c*. 0.10 g cm^−3^ in *Jacaratia spinosa* (Aubl.) A.DC) to species, such as lignum vitae (*Guaiacum sanctum* L.), whose wood is denser than water (*c*. 1.05 g cm^−3^) and therefore sinks at any moisture level. However, wood density also varies within and among individuals of the same species (Anderegg *et al*., 2021; Fajardo *et al*., 2022; Yang *et al*., 2023). Intraspecific variation in traits provides an imprint of how organisms react to changes in their environment through adaptation and morphological plasticity (Bolnick *et al*., 2011; Moran *et al*., 2016; Girard‐Tercieux *et al*., 2023) and can play an important role in ecosystem functioning (Des Roches *et al*., 2018). Better knowledge of shifts with ontogeny and along environmental gradients would improve vegetation models (Berzaghi *et al*., 2020), predict how species ranges shift in response to climatic change and disturbance (Anderegg & HilleRisLambers, 2016), and create more robust wood density maps for assessment of functional diversity and vegetation carbon stocks (Boonman *et al*., 2020; Sæbø *et al*., 2022).

Radial changes within tree trunks and branches are a common source of intraspecific variation in wood density, usually interpreted as a reflection of hydraulic and mechanical changes during ontogeny (Wiemann & Williamson, 1988; Woodcock & Shier, 2002). In plant species with low wood density near the pith, wood density often increases towards the outer trunk layers, while the opposite may occur in plants with high near‐pith wood density (Woodcock & Shier, 2002; Hietz *et al*., 2013; Plourde *et al*., 2015; Longuetaud *et al*., 2017; González‐Melo *et al*., 2022), although there are many exceptions to this pattern (Osazuwa‐Peters *et al*., 2014; Bastin *et al*., 2015). A link between radial variation in wood density and plant ecological strategies has also been suggested: pioneer plants have low density wood and grow fast early on, but invest in denser tissues as individuals mature. By contrast, shade‐tolerants build dense tissues initially, but may invest more in diameter growth than tissue density when reaching the canopy (Woodcock & Shier, 2002; Bastin *et al*., 2015). However, we do not know how consistent and important these patterns are at global scales. Radial changes in wood density do not always map onto ecological strategies (Hietz *et al*., 2013), may be influenced by deposition of chemical compounds in heartwood (e.g. nonstructural, secondary metabolites known as ‘extractives’, Lehnebach *et al*., 2019) and vary among conspecific individuals or even within individuals (Osazuwa‐Peters *et al*., 2014).

Wood density also varies along the hydraulic pathway and across plant organs within an individual, another source of intraspecific variation (Schuldt *et al*., 2013; Longuetaud *et al*., 2017; Momo *et al*., 2020). In trees, for example, wood density has been hypothesized to increase from trunks to branches, because high density should provide more benefits to mechanical stability in horizontal branch than vertical trunk wood (Anten & Schieving, 2010; van Casteren *et al*., 2012). However, while branch and trunk wood densities are generally tightly correlated with one another, there is little agreement on whether branches are more (Fegel, 1941; Dibdiakova & Vadla, 2012; Fajardo, 2018; Billard *et al*., 2020) or less dense than trunks (Swenson & Enquist, 2008; Sarmiento *et al*., 2011; He & Deane, 2016), and there is also variation within trunks and branches (Schuldt *et al*., 2013; Terrasse *et al*., 2021). A confounding factor may be that wood density varies less in branches than in trunks: data from temperate ecosystems show that wood density increases from trunk to branches in species with low‐density trunk wood and shows the opposite pattern in species with high‐density trunk wood (MacFarlane, 2020). It is unclear whether this pattern generalizes across biomes and also whether it reflects different functional requirements of branches and trunks. However, it has been hypothesized that trunk‐branch gradients simply reflect radial ontogenetic patterns between juvenile and mature wood, since more distal organs are younger and contain higher fractions of sapwood (Gartner, 1995).

Wood density also varies across individuals of the same species due to differences in genetics and environments (Zobel & van Buijtenen, 1989). As environmental conditions become more extreme (drier, less fertile, more shaded and more windy), species are expected to grow more slowly and invest more resources in dense and stress‐resistant tissues (Chave *et al*., 2009). Similar effects are expected within species (Anderegg *et al*., 2021). For example, individuals that build tissues with narrow conduits and thick fibre and conduit walls should be more resistant to embolism (Hacke *et al*., 2001; Olson *et al*., 2020). By contrast, in warm, fertile and frequently disturbed environments with high plant turnover, fast‐growing individuals with low wood density are expected to be more competitive and successful (Muller‐Landau, 2004; Yang *et al*., 2023). However, genetic control over wood density is high, suggesting that the variation among individuals of the same species is limited (Zobel & Jett, 1995). Wood density variation may also be limited by covariation with other traits and trade‐offs between different wood functions (Ziemińska *et al*., 2013; Anderegg & HilleRisLambers, 2016). For example, low‐density wood can sometimes be beneficial even in harsh conditions, as low‐density species tend to have greater water storage and capacitance (Ziemińska *et al*., 2020), a potential advantage in dry environments.

Overall, theory, empirical observations and common sense predict that wood density varies predictably within species. However, while many studies find that intraspecific variation is predictable (Anderegg & HilleRisLambers, 2016; Anderegg *et al*., 2021; Farias *et al*., 2023), just as many do not (Richardson *et al*., 2013; Fajardo, 2018; Rosas *et al*., 2019; Umaña & Swenson, 2019). Intraspecific variation is generally smaller than interspecific variation (Osazuwa‐Peters *et al*., 2014), so it is easily confounded with measurement errors and methodological differences in how wood density is determined (Barbosa & Fearnside, 2004; Williamson & Wiemann, 2010; Jati *et al*., 2014; Vieilledent *et al*., 2018). To date, the largest global wood density collections, including the GWDD v.1 (Zanne *et al*., 2009), do not systematically record the tissue types and plant organs where measurements were taken. As a result, there is a fundamental lack of knowledge about the extent of intraspecific variation in wood density and its determinants within and among individuals, with substantial implications for ecological models and carbon estimates in woody ecosystems (Nogueira *et al*., 2007; Momo *et al*., 2020). In particular, we lack practical guidelines as to when to exhaustively measure it vs when it can be safely ignored.

Here, we introduce a substantially updated and improved version of the Global Wood Density Database (GWDD v.2), which more than doubles the taxonomic coverage of the original database (from 7555 to 16 829 taxonomically resolved species), increases the number of records from 16 468 to 109 626, and, as available, includes a detailed description of where and how measurements were taken within and across individuals. Using these data, we addressed the following questions: (1) How large is intraspecific variation in wood density? (2) How much of this intraspecific variation can be explained by differences in wood density among plant organs? (3) How much of intraspecific variation can be explained by environmental factors, such as temperature, water deficit, wind speeds and soil fertility? Based on our results, we also (4) make a number of recommendations to effectively incorporate variation among and within individual plants into models to improve predictions of wood density.

## II. Materials and Methods

### 1. Assembling the Global Wood Density Database v.2

The GWDD v.2 is a substantial update of the GWDD v.1, an open‐access database that consisted of a list of 16 468 tissue density values from *c*. 7500 species. It was assembled from > 200 sources and included a taxonomic identifier, a region where the measurement was taken, and a literature reference (Chave *et al*., 2009; Zanne *et al*., 2009). Since its publication, many new wood density values and large trait databases have been published, and improved methodologies have been developed to improve consistency across studies (Williamson & Wiemann, 2010; Vieilledent *et al*., 2018; Langbour *et al*., 2019; Farias *et al*., 2020; Radtke *et al*., 2023; Cuny *et al*., 2025). We used this as an opportunity to expand the database and create a new, improved version.

First, we critically re‐examined the original database and updated 42% of entries (*n* = 6968, details in Supporting Information Methods S1). Second, we included additional wood density measurements from published and unpublished sources (Methods S1, S2). We put particular emphasis on previously undersampled biomes, such as dry forests, savannas and the species‐rich tropics. We also included individual measurements instead of aggregated values and created comprehensive documentation. The new database contains 45 attributes that report the original values, sampling techniques and data transformation methods (Table S1). The database is available online on Zenodo (doi: 10.5281/zenodo.16919509).

#### Wood density definition and conversion factors

In the GWDD v.2 and throughout this study, wood density is defined as ‘basic’ wood density, the mass of an oven dried wood sample divided by its fresh (or water‐saturated) volume (g cm^−3^). Wood density thus measures the dry mass contained in the wood volume of live plants and is an indicator of a plant's investment in woody tissues. When normalized by the density of water (1 g cm^−3^), it is also referred to as ‘wood specific gravity’ (g g^−1^; Williamson & Wiemann, 2010), but throughout our analyses, we use the term ‘wood density’. When assembling the GWDD v.2 and in all following analyses, we also included the tissue densities of tree‐like monocots without secondary growth, as this was consistent with common inventory protocols (Condit, 1998; The SEOSAW Partnership, 2021) and global tree databases (e.g. Beech *et al*., 2017). In total, monocots contributed 186 records from 91 species most of which were either Arecaceae (65) or Asparagaceae (18). Since these monocots amounted to < 0.2% of the total records, their inclusion had a negligible effect on results.

Different wood density definitions are available in the literature. Green density, the fresh mass of wood divided by fresh volume, reflects actual plant growing conditions (Niklas & Spatz, 2010). In the timber industry, a relevant quantity is air‐dry wood density, the mass of wood divided by its volume, with both measured at ambient air moisture (*c*. 10–15%), reflecting properties of wood in the conditions in which it is used (Détienne & Chanson, 1996; FPL, 1999). Oven‐dry density, that is dry mass over dry volume, has also been reported in the literature (Deklerck *et al*., 2019), and dendrochronological studies often derive correlates of wood density variation within and between tree rings with X‐ray techniques (Jacquin *et al*., 2017). Fortunately, air‐ and oven‐dry densities can each be converted into basic density through physical conversion factors (Sallenave, 1971; Brown, 1997; Ilic *et al*., 2000), and recent research has shown that this can be done with as little error as 0.015 g cm^−3^ (*c*. 2.5% of typical mean wood density, Vieilledent *et al*., 2018). These factors were also applied in the construction of the GWDD v.2 to maximize the taxonomic and geographic coverage of wood density (Methods S3). Converted values and the source quantity were recorded in the columns *value_reference* and *quantity_reference*, the conversion factor in *wsg_conversion*, and the derived basic wood density value as *wsg* (‘wood specific gravity’).

#### Aggregation levels

The GWDD v.2 provides extensive information on where and how records were obtained, which was not available in the GWDD v.1. These new variables include *site*, an informal description of the measurement site, *longitude* and *latitude* in the decimal system, and *country*. The attribution to a *region* has been revised since GWDD v.1 to better reflect geographical variation (Table S1). The database also contains information on sample type (*type_sample* for ‘core’ or ‘disc’), measurement location within plants (*location_sample* for ‘trunk’, ‘branch’ or ‘root’, for example, and *type_tissue* for ‘heartwood’, ‘sapwood’ or ‘bark’), whether a particular wood density value is the mean value of multiple individual plants (*plant_agg*), and how many individuals were aggregated (*plants_sampled*). If several measurements were available for the same individual, they were recorded with the same *id_plant*. Direct estimates of variation around mean values were not included in the GWDD v.2, as they were not consistently reported in the literature. Where detailed measurement information was not available, attributes were left empty (‘NA’). These samples were excluded from analyses of intraspecific wood density variation in this study.

#### Taxonomic name resolution

Taxonomic names were newly standardized via the worldflora R package (Kindt, 2020) and the June 2023 version of the *World Flora Online* (WFO) database (The World Flora Online Consortium *et al*., 2023). Taxon names were converted in the field *species_reference_canonical*, including infraspecific assignations (e.g. variety, subspecies, hybridization) and resolved via the default fuzzy matching in the worldflora package. Taxonomic authorities were not included as inputs for the matching, as they were inconsistently reported in the source data. For unmatched taxa and anything beyond a missing, added or switched letter, the matching was repeated without infraspecific assignations. In the case of multiple matches, we chose the default value provided by *WFO* (called ‘smallest id’). Any remaining taxa were manually corrected. We also extracted information on plant families from the WFO database and reported it in the GWDD v.2. Overall, only 34 entries (22 genera) could not be matched to any family.

Taxonomies are constantly updated to resolve ambiguities in species definitions, and sometimes, taxonomic resolution leads to a reclassification of species as subspecies and varieties (or vice versa). For simplicity, we recorded the entire scientific name provided by worldflora in the GWDD v.2's *species* column, including infraspecific epithets. We also used this species definition for analyses in our study to provide the most conservative estimates of intraspecific variation. However, preliminary tests revealed that only a negligible portion of the analysed species had infraspecific epithets (*c*. 1% of all species with individual level wood density records, < 1% among high‐quality records), so this choice had no discernable effect on results.

### 2. Intraspecific wood density variation

#### Coverage of taxonomic and geographic variation

Overall, we assembled > 100 000 records from *c*. 17 000 plant species in the GWDD v.2. We assessed the representativeness of the species included in the GWDD v.2 with regard to the number of woody taxa world‐wide estimated by assuming that 45% of the flowering plants are woody (FitzJohn *et al*., 2014), and the total number of flowering plants is *c*. 400 000 (Enquist *et al*., 2019). We also used a verified list of known tree species (globaltreesearch 1.7; Beech *et al*., 2017), resolved via *WFO* for consistency, to assess which percentage of tree species in each country had a corresponding wood density estimate in the GWDD v.2. Intraspecific coverage was assessed through the number of records per species.

#### Statistical analysis of intraspecific wood density variation

To assess the extent and drivers of intraspecific wood density variation, we examined measurements from multiple sites per species, multiple individuals per site and multiple measurement locations per individual. For some species, the database contains multiple samples of individuals, but only from a single site, while for others, the database contains samples from multiple sites, but each with a single individual. To address this issue, we created subsets of the GWDD v.2 for each question and alternative modelling strategies to ensure robustness of results (see Table S3 for an overview of models and subset sizes). Throughout, ‘intraspecific variation’ refers to cases where biological variation can be confidently separated from measurement errors. For example, tissue type (heartwood/sapwood) and environmental factors should affect biological variation, not measurement error. By contrast, when variation cannot be attributed to a specific factor, intraspecific variation plus measurement error are referred to as the ‘(residual) wood density distribution’.

Across all datasets, we removed records not identified to species level (‘genus’ in the *rank_taxonomic* column of the database), samples consisting of bark (‘bark’ in the *type_tissue* column of the database) and records from experiments (identified by the words ‘fertilizer’ or ‘treatment’ in *experiment_design*) or from plantations (recorded as ‘plantation’ in *type_forest*). We also excluded root samples (‘root’ in the *location_sample* column) due to small sample sizes.

Unless otherwise stated, all datasets were analysed using mixed effects models with varying random slopes and intercept terms and qualitative explicative variables (e.g. ‘sapwood’ vs ‘heartwood’) coded as numerical variables (0, 1). Models were fitted in the R environment (R Core Team, 2023), both with a Bayesian approach – using the brms package (Bürkner, 2018) and STAN (Carpenter *et al*., 2017) – and the maximum likelihood framework of the lme4 package (Bates *et al*., 2015, ‘bobyqa’ optimizer). The Bayesian approach was the default, due to flexible model construction, regularization through priors and full propagation of uncertainty. Models were assessed for convergence using standard diagnostics (*R*‐hat ≤ 1.02) and posterior predictive checks (further details in Methods S4; Table S3; Fig. S1). Throughout, we also report lme4 estimates, as they are less expensive computationally and thus more readily used in practice, especially when relying on large databases. Model fits were assessed by comparing measured and fitted values via root mean square error (RMSE, g cm^−3^) and *R* ^2^. A complete list of R packages used in the analysis can be found in the Methods S5.

#### Quantification of intraspecific variation in wood density

To assess the overall extent of intraspecific variation in wood density, we first partitioned total wood density variance and its components. We fitted a model with random effects for species nested within genera, genera nested within families and a crossed random effect for methodological bias (the bibliographic reference or ‘source’, Model M1, Table S3). We computed variance as the sum of variances across levels plus residual variance. To assess robustness, we also fitted a separate model without the ‘source’ effect (Table S3, M2) and restricted the analysis to species with greater than or equal to three individuals per species, greater than or equal to three species per genus and greater than or equal to three genera per family (*n* = 49 991, *n*_species_ = 2735; Models M3–M4).

Second, to assess the contribution of different sources of intraspecific variation to its overall extent, we partitioned the intraspecific variance of wood density. We did so by fitting models with random effects for individuals nested within sites, and sites nested within species, each time with and without a crossed effect for measurement source (formulas in Table S3, M5–M8). We restricted the analysis to subsets of species present in at least *k* sites, one site with at least *k* individuals and one individual with at least k measurements (first setting *k* = 2 and then repeating for *k* = 3). Sites were defined as the collection of data within the same 1 km^2^ grid pixel. This approach mirrored the resolution of the climate input data and accounted for geolocation uncertainty (e.g. rounding of longitude and latitude values to 2 decimals). Data subsets comprised *n* = 19 246 (*n*_individual_ = 14 373, *n*_site_ = 1270, *n*_species_ = 147) records for *k* = 2, and *n* = 2494 (*n*_individual_ = 1052, *n*_site_ = 233, *n*_species_ = 35) for *k* = 3.

The shape of intraspecific wood density distributions was assessed through the direct modelling of the residual variance parameter σ (Table S3, Model M1, cf Methods S4) and by comparing how well normal and lognormal distributions fitted each species’ wood density distribution (Shapiro–Wilk test for untransformed vs log‐transformed wood densities with *P* < 0.05). To account for methodological differences and report biological variation, we always subtracted the random ‘source’ effect before reporting wood density variation.

#### Estimating variation in wood density within individuals

Variation in wood density within individuals was assessed by subsetting to species with measurements for both sapwood and heartwood (*n* = 679, *n*_species_ = 150), or for both trunkwood and branchwood (*n* = 48 494, *n*_species_ = 2018). The fitted models (Table S3, M9–M10) had fixed effects for either sapwood (0 for heartwood, 1 for sapwood) or branchwood (also 0/1), random intercepts and slopes at the species level, and a crossed random effect for measurement source. To assess whether wood density followed predictable gradients from the pith outwards to the bark and from the trunk upward to the branches, we calculated species means for each woody component and checked whether the slopes of major axis regressions (lmodel2 package; Legendre, 2024) of sapwood vs heartwood and branchwood vs trunkwood densities were < 1 (indicating a decrease) or > 1 (indicating an increase, Table S3, M11–M12).

To assess robustness and potential confounding effects, such as higher sapwood fraction in branches or variation in sample sizes among taxa, we repeated the analysis with a subset of branchwood and trunkwood densities taken entirely from sapwood (*n* = 1193, *n*_species_ = 523, M13), with a higher‐quality dataset (species with ≥ 5 measurements both for branches and trunks, selecting five random measurements from each, *n*_species_ = 189, M14), and a subset of records where both branch and trunk samples were taken from the same individuals (*n* = 3527, *n*_species_ = 145, M15, fitted at individual plant level, taking a random sample from both trunk and branch).

#### Environmental predictors of intraspecific wood density variation

To examine wood density variation across environmental gradients, we used species sampled from at least two sites and paired them with the following bioclimatic layers that represent multiple axes of plant environmental gradients, including extremes: annual mean temperature (°C), site water balance (kg m^−2^ yr^−1^, but with the sign reversed to indicate water deficit), and mean wind speed (m s^−1^) at *c*. 1 km resolution (30 arcseconds). We not only used the data from the CHELSA/BIOCLIM+ climatology 1981–2010 (Karger *et al*., 2017; Brun *et al*., 2022) but also repeated the analysis with coarser products from the TerraClimate climatology 1981–2010 (1/24° or *c*. 5 km at the equator), relying on climatic water deficit (mm) instead of site water balance (Abatzoglou *et al*., 2018). While site water balance is a general measure of the availability of water to plants (Brun *et al*., 2022), climatic water deficit directly measures drought stress as the difference between potential and actual evapotranspiration. We also included the following soil layers: sand fraction (g kg^−1^), pH (unitless) and cation exchange capacity (in mmol(c) kg^−1^), based on the *soilgrids* product (Hengl *et al*., 2017). By default, we report only effects that remained qualitatively consistent across analysis methods (no shift in sign). For simplicity, effect sizes are always taken from the full dataset using CHELSA/BIOCLIM+ predictors.

We matched predictors to wood density observations and assessed environmental effects both among and within species with the following model (cf Table S3, M16–M19): $wd_{\mathrm{ij}}=\beta_{0}\;+\;\sum_{k=1}^{n}\beta_{k}\cdot \mathrm{en}v_{\mathrm{kj}}\;+\;\sum_{k=1}^{n}\gamma_{k}\cdot \Delta \mathrm{en}v_{\mathrm{kij}}\;+\;\gamma_{0j}+\sum_{k=1}^{n}\gamma_{\mathrm{kj}}\;\cdot \Delta \mathrm{en}v_{\mathrm{kij}}+\;\varepsilon_{\mathrm{ij}}$, with $\varepsilon_{\mathrm{ij}}\sim N\left(0,\sigma^{2}\right)$. Here, *n* is the number of individuals, $wd_{\mathrm{ij}}$ is the wood density of individual *i* belonging to species *j*, $\mathrm{en}v_{\mathrm{kj}}$ is the environmental variable *k* averaged across all individuals from species *j*, and $\Delta \mathrm{en}v_{\mathrm{kij}}$ is the same environmental predictor *k*, but group‐centred around the species mean value, and $\varepsilon_{\mathrm{ij}}$ is the error. The model thus partitions environmental effects into interspecific effects, where $\mathrm{en}v_{\mathrm{kj}}$ is the typical environment of species *j*, and intraspecific effects, where $\Delta \mathrm{en}v_{\mathrm{kij}}$ represents how much the individual *i* deviates from the species mean environment. The model is equivalent to a standard linear‐mixed effects model with random slopes and intercepts for species, but with added group‐level predictors $\mathrm{en}v_{\mathrm{kj}}$ that control for predictable variation among species (Table S3, models M16–M17, Bafumi & Gelman, 2007). $\beta_{0}$ is the overall intercept, $\beta_{k}$ and $\gamma_{k}$ are fixed‐effect parameters, and $\gamma_{0j}$ and $\gamma_{\mathrm{kj}}$ are random intercept and slope parameters for species *j*, respectively. We chose this model as it allows for partitioning of intra‐ and interspecific effects and is more robust when predictors vary systematically with grouping factors (Bafumi & Gelman, 2007).

Since some species may cover only a narrow environmental range, we repeated the analysis with a subset of species for which at least one environmental factor covered a large range (Table S3, *n* = 30 128, *n*_species_ = 692, M18–M19), defined via TerraClimate and *soilgrids* as the 90^th^ percentiles of all species' ranges (Δ_Temperature_ ≥ 8°C, Δ_Water Deficit_ ≥ 450 mm, Δ_Wind speed_ ≥ 1.8 m s^−1^, Δ_Sand content_ ≥ 300 g kg^−1^, Δ_pH_ ≥ 1.5 or Δ_Cat. exch. cap._ ≥ 165 mmol(c) kg^−1^). To assess the consistency of predictors across biomes, we fitted separate models for tropical species (≥ 3 measurements in the tropics, *n* = 8783, *n*_species_ = 700, M20–M21) and extratropical species (≥ 3 measurements outside the tropics, *n* = 26 437, *n*_species_ = 247, M22–M23).

In all models, intra‐ and interspecific effects were examined on the same standardized scale. However, as environmental gradients among species were larger than among individuals of the same species, we also tested the rescaling of effect sizes to realized environmental ranges for the tropical and extratropical subsets, that is standardizing *after* separation of intra‐ and interspecific effects by their respective SD. We fitted additional models to gymnosperms only (Table S3, *n* = 12 089, *n*_species_ = 59, M24–M25) to assess the stability of global effects when sampling is reduced to a small number of anatomically diverging taxa.

#### The effect of intraspecific variation on wood density predictions

Since wood density measurements are destructive, samples are usually only taken from a subset of plants, from nearby conspecifics or from global databases. The samples are then used to predict the wood density of the remaining individuals, for example, by using species mean values, or, if those are not available, the average of wood densities from the same genus or plot (Flores & Coomes, 2011; Réjou‐Méchain *et al*., 2017). If many traits have been measured, more complex imputation methods are available (Schrodt *et al*., 2015). However, most methods risk confounding intraspecific and interspecific variation and it is unclear what to do in edge cases, for example if one to two measurements from the same species are available, is it better to estimate an individual's wood density by: (1) directly using these values and averaging them; (2) attributing the genus mean value; or (3) combining both types of information, for example through taxonomic or phylogenetic hierarchical modelling? It is also (4) unclear whether local measurements should be weighted more strongly to account for environmental gradients in wood density.

To answer these questions, we first assessed the influence of intraspecific variation on species‐level wood density estimates. We computed average wood densities for all species with > 5 measurements (*n*_species_ = 1667) and assessed how accurately these reference values could be predicted if species were not well sampled. To do so, we cycled through all 1667 species, in turn removed either all species‐specific measurements or all species‐specific measurements except one or two randomly chosen ones, and then estimated the species' mean wood density from the remaining data. The estimation was carried out with three models: (1) the default approach of estimating wood density means from a genus average (when no species‐specific measurements exist) or a species average (when one or two species‐specific measurements exist); (2) a hierarchical model of wood density that included a nested taxonomic structure (*family*/*genus*/*species*) and nested random effects for sites within studies; and (3) the same model as in (2), but with an optional fixed effect for trunkwood vs branchwood (Table S3, models M26–M27). Model performance was estimated via RMSE (g cm^−3^) and *R* ^2^ (Table S16).

Second, we tested how accurately we could predict the wood density of an individual plant depending on how well the species was sampled locally. To do so, we selected species with measurements from at least three sites, and where at least four of its individuals were measured at each of the three sites (*n*_species_ = 318). For each of the individuals, we then reduced the set of locally measured conspecifics to 0, 1, 2 or 3 (selecting random individuals where possible) and for each case tested how well the individual's wood density could be inferred from the remaining data. We tested five models: (1) a species average across the entire dataset; (2) a species average, but using only values measured as part of the same study; (3) a species average, but using only values measured locally; and (4, 5) the same hierarchical models as described above (Table S3, M26–M27). Model performance was estimated via RMSE (g cm^−3^) and *R* ^2^ (Table S17).

## III. Results

### 1. The Global Wood Density Database

To create the GWDD v.2, we assembled 109 626 wood density records from 166 countries, 617 primary sources and 17 262 taxa across all woody biomes and biogeographic realms. Of these taxa, we resolved 16 829 to species level. We also included information on aggregation levels, conversion factors and precise geographic location, as well as an additional 15 093 bark density records from 57 studies. The fully assembled GWDD v.2 contained 6.7× as many wood density records and 2.3× as many species as the GWDD v.1 (16 468 records across 7453 accepted species). We estimated that the GWDD v.2 covered 10% of all woody species, 24% of all known tree species and 49% of gymnosperm species. Of the families with the most known tree species, Sapotaceae and Fabaceae were best represented in the database (38% and 34% coverage). By contrast, wood density estimates were rare in Arecaceae (6%, not true wood), Araliaceae (10%) and Melastomataceae (12%).

**Fig. 1:**
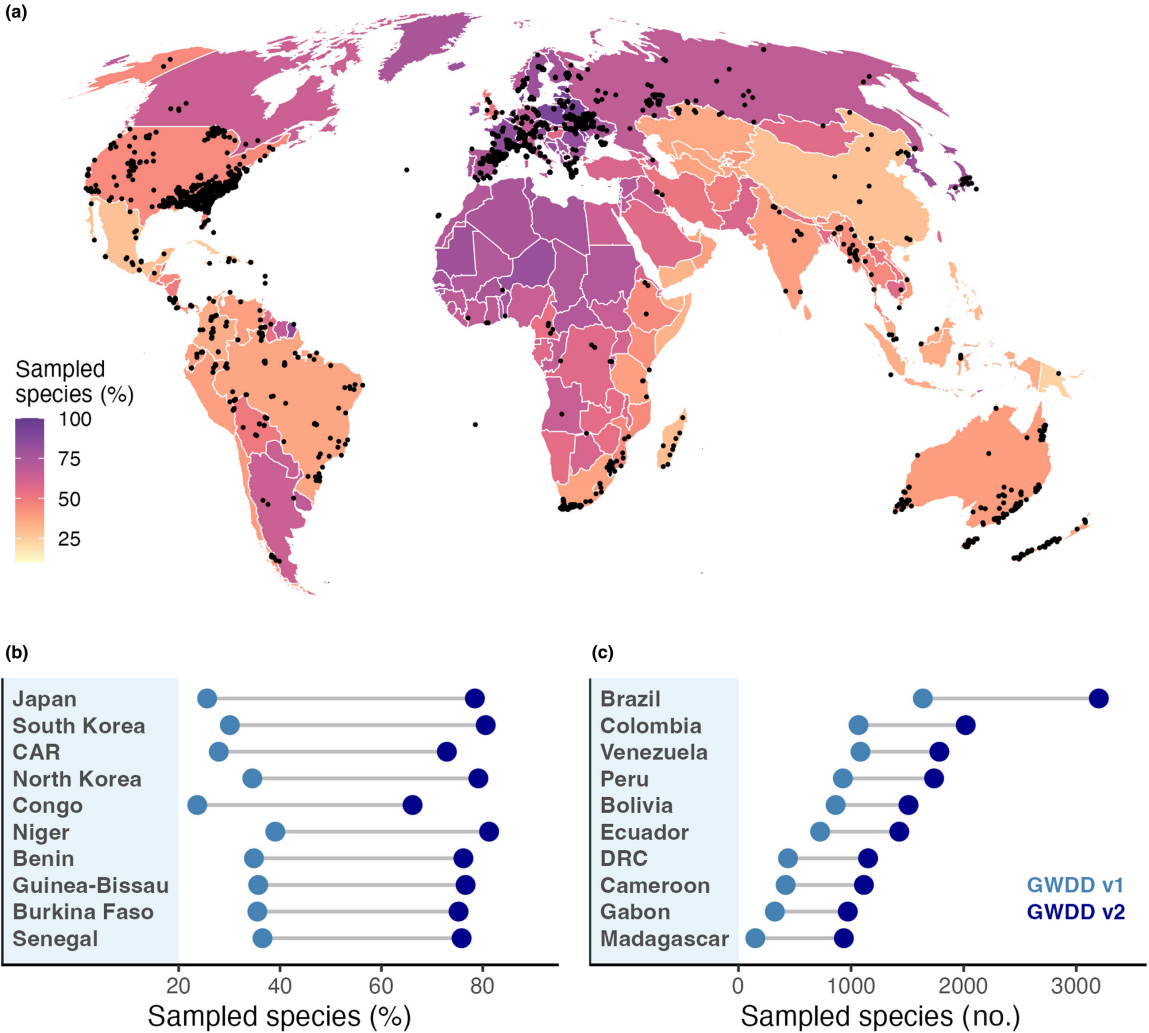
Tree species coverage in the Global Wood Density Database (GWDD) v.2. Panel (a) maps the percentage of each country's known tree species matched to the GWDD v.2 (background colour gradient), with explicitly geolocated wood density measurements overlaid (black dots). Not all measurements are geolocated, so countries can have high species coverage despite a lack of country‐specific measurements (e.g. West African countries). Conversely, countries can have many geolocated records in temperate systems, but low species coverage due to poor coverage in subtropical or tropical zones (e.g. the United States). Country borders follow *Natural Earth* (https://www.naturalearthdata.com/, last accessed 30 Oct 2025). Panel (b) shows the top 10 countries with the largest improvement in species coverage (%) between GWDD v.1 and GWDD v.2. Panel (c) shows the top 10 countries with the largest improvement in sampled species, in descending order of GWDD v.2 coverage.

Out of all the GWDD v.2's records, 83502 (76%) were provided at individual plant level, and 58 675 entries (54%) were precisely geolocated. For 4508 species, at least five wood density measurements were available, and combinations of branch‐trunk wood measurements were available for 2018 species (146 families). For 78 species, mostly in Pinaceae and Fagaceae, there were > 100 wood density measurements. The majority (65%) of the wood density values were directly measured as basic wood density (71 746). The remaining values were converted from air‐dry (*c*. 29%) and oven‐dry wood densities (*c*. 6%). Geographic coverage varied widely, from a near‐complete coverage of recorded tree species in high latitudes to < 50% in tropical regions (95% range: 27.3%; 98.5%, Fig. 1a). We found the strongest improvements in coverage in East Asian and West African countries, from 30 to 40% in the GWDD v.1 to *c*. 80% in the GWDD v.2 (Fig. 1b). The strongest increases in absolute species numbers (Fig. 1c) were recorded in tropical countries in South America and Africa. For example, we included wood density records for 1563 new species in Brazil, nearly doubling the GWDD v.1's species coverage (1637), and 786 new species in Madagascar, adding > 5× the amount of species recorded previously (150).

### 2. The extent and shape of intraspecific variation in wood density

Globally, wood density displayed a normal distribution with a mean of 0.56 g cm^−3^ (SD = 0.178 g cm^−3^). The majority of this variation (77%, SD = 0.156 g cm^−3^) was accounted for by variation at the taxonomic family (30%), genus (30%) or species levels (17%), with the rest attributed to study methodology (8%) or intraspecific and unknown variation (15%; 0.068 g cm^−3^, Tables 1, S4; Figs S2–S3). The intraspecific contribution was robust to model details (Tables 1, S4–S5). For well‐sampled taxa, we partitioned intraspecific variance and found that wood density variation among sites exceeded variation among individuals within sites (SD = 0.025–0.042 g cm^−3^ vs SD = 0.017–0.028 g cm^−3^, Table S6), but was small overall (*c*. 20–30% of total intraspecific variation) and smaller than residual variation (variation within individuals + unknown measurement error, SD = 0.040–0.045 g cm^−3^, Tables S5–S6).

**Table 1:** Partitioning of wood density variance.

	M1: Full dataset (incl. source effect)		M2: Full dataset (no source effect)		M3: High‐quality subset (incl. source effect)		M4: High‐quality subset (no source effect)	
**Wood density (g cm** ^**−3**^ **)**	*Estimate*	*CI*	*Estimate*	*CI*	*Estimate*	*CI*	*Estimate*	*CI*
(Intercept)	0.557	[0.540–0.575]	0.554	[0.538–0.569]	0.579	[0.554–0.605]	0.575	[0.552–0.599]
**Random effects**	*Estimate*	(*%Var*)	*Estimate*	(*%Var*)	*Estimate*	(*%Var*)	*Estimate*	(*%Var*)
Family	0.098	(30%)	0.098	(32%)	0.067	(17%)	0.065	(17%)
Family : genus	0.097	(30%)	0.098	(32%)	0.092	(33%)	0.094	(36%)
Family : genus : species	0.074	(17%)	0.077	(20%)	0.077	(23%)	0.079	(26%)
Source	0.049	(8%)			0.049	(9%)		
Residual (σ)	0.068	(15%)	0.071	(17%)	0.068	(18%)	0.071	(21%)

Estimates of variance partitioning from four models. All models partition wood density variance by hierarchically nesting taxonomic family, genus and species levels, but differ in whether they also account for systematic measurement errors (approximated by a random ‘source’ effect, models M1/M3 vs M2/M4) and whether they use the full dataset (M1 and M2, *n* = 41 879, *n*_species_ = 2160) or a higher‐quality subset (M3 and M4, *n* = 30 128, *n*_species_ = 692). Point estimates of the wood density intercept are posterior means (‘Estimate’, g cm^−3^), intervals 95% credibility intervals (‘CI’). Variance estimates are provided as random effect SD in units of wood density (‘Estimate’, g cm^−3^) and proportion of total variance (‘%Var’, in brackets). Residual variation includes random measurement errors and thus provides an upper bound on intraspecific variation (15–21%). Further model details can be found in Supporting Information Table S3, and an extended version in Table S4.

Intraspecific variation differed in extent and shape between species, with a few species varying much more than the others (e.g. SD = 0.094 g cm^−3^ for *Quercus ilex* L., compared with SD = 0.038 g cm^−3^ for *Quercus alba* L.; Figs S3, S4). The distribution of wood density values of conspecifics was generally heavy‐tailed, but there was no clear signal of skew, with the lognormal distribution more often rejected than the normal distribution (13.7% vs 12.6%, Shapiro‐Wilkes *P* < 0.05 for log‐transformed and untransformed values).

### 3. Wood density variation within individuals

**Fig. 2:**
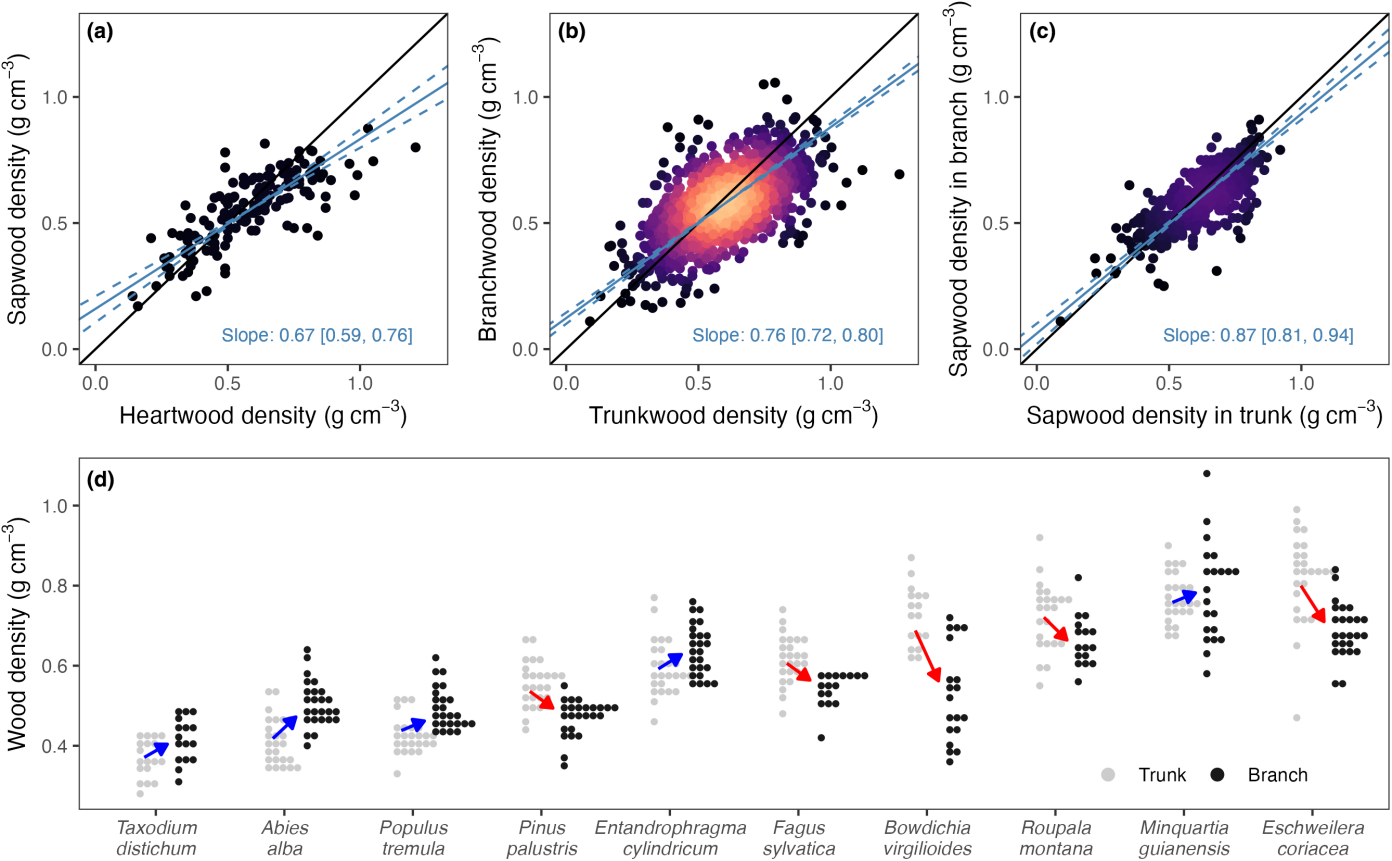
Variation in wood density across plant tissue types. Scatterplots of species means for densities of (a) heartwood vs sapwood, (b) trunkwood vs branchwood and (c) trunkwood vs branchwood when measured in sapwood only. Every dot represents a species, blue lines are Major Axis regression lines, *dashed* lines their 95% confidence intervals, with 1 : 1 lines in solid black. Areas with high point densities are coloured brightly. The corresponding slope estimates, with confidence intervals in square brackets, are provided in the bottom right corner. Panel (d) shows the distribution of trunk and branch wood densities for 10 species, ordered by increasing trunk wood density. For visualization, we selected a maximum of 25 random samples for each species and trunk/branch combination. Arrows indicate increases (blue) and decreases (red) in the median wood density from trunk to branch.

Across plant tissue types, there were strong correlations between heartwood and sapwood densities (Pearson's *r* = 0.78; Fig. 2a) and between trunkwood and branchwood densities (*r* = 0.67; Fig. 2b; Table S9). However, slopes were < 1 in both cases. There was a 50% reduction in the variance of sapwood compared with heartwood (0.144 vs 0.197 g cm^−3^) and a *c*. 30% reduction in the variance of branchwood compared with trunkwood (0.120 g cm^−3^ vs 0.145 g cm^−3^). When comparing only sapwood samples from trunks and branches, this effect was weaker (0.112 vs 0.124 g cm^−3^, less than a 20% reduction in variance) and the correlation was stronger (*r* = 0.76, *n* = 523; Fig. 2c; for more analyses see Table S9; Figs S5, S6). There was a weak average decrease in wood density from heartwood to sapwood of −0.001 g cm^−3^ and a stronger, but still small decrease of −0.023 g cm^−3^ from trunkwood to branchwood (Table S8). However, both effects varied strongly among species (slope SD = 0.060 g cm^−3^ for heartwood vs sapwood and SD = 0.075 g cm^−3^ for trunkwood vs branchwood). Wood density increased from trunk to branch in low‐density species, such as gymnosperms or temperate angiosperms (+0.09 g cm^−3^ or *c*. 22% in *Abies alba* Mill.), while wood density decreased from trunk to branch in high‐density species, typically found in the tropics (−0.13 g cm^−3^ or −16% in *Eschweilera coriacea* (DC.) S.A.Mori), but many exceptions existed and patterns were noisy (Fig. 2d).

### 4. Wood density variation among individuals

**Fig. 3:**
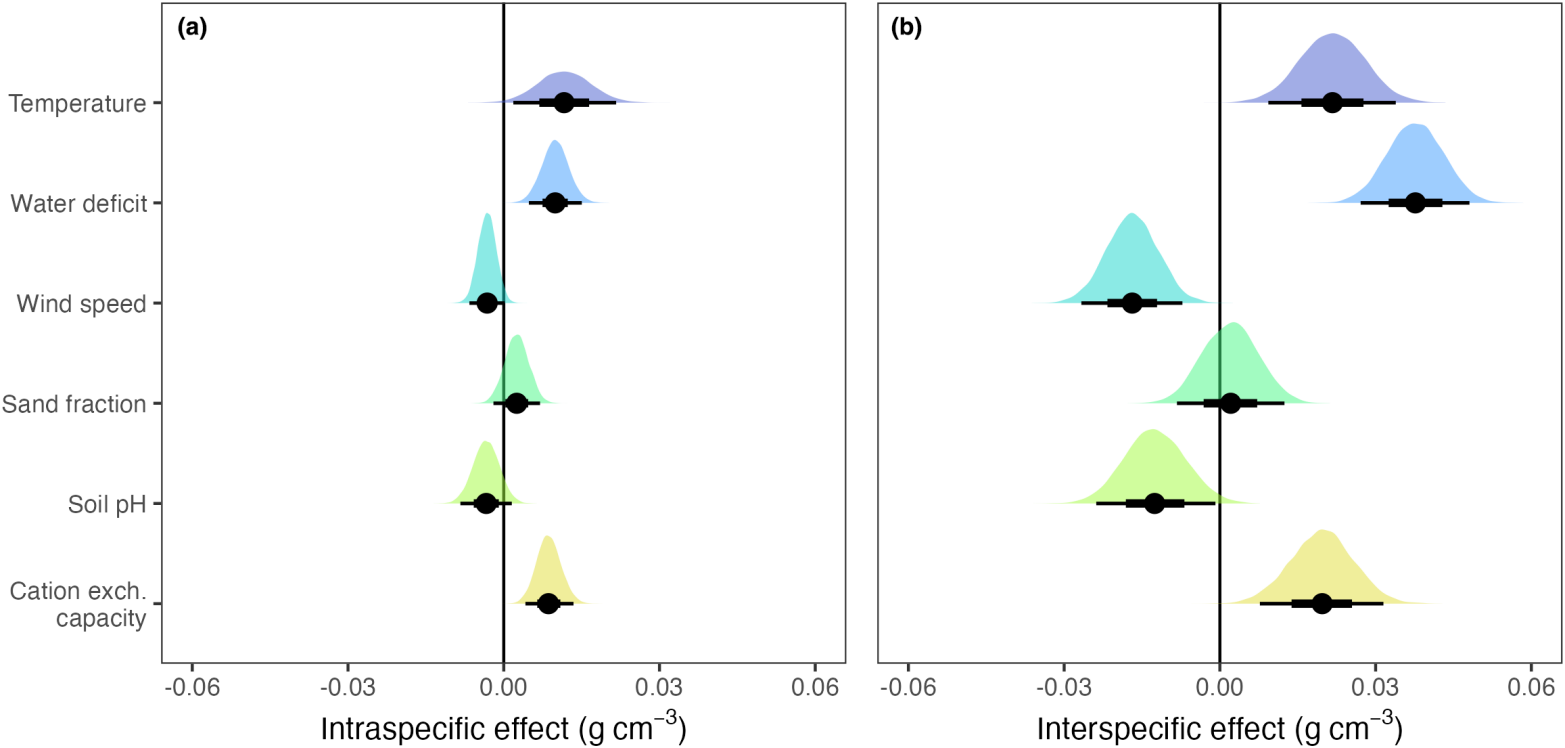
Environmental predictors of wood density. Shown are the global effects of climatic and edaphic predictors on wood density, separated into intraspecific (a) and interspecific effects (b). Estimates of effect sizes were derived from a Bayesian hierarchical model, with all predictors scaled by one SD (Model M9, cf Supporting Information Tables S3, S10). Climatic variables were from CHELSA/BIOCIM+ (Karger *et al*., 2017; Brun *et al*., 2022), edaphic variables from *soilgrids* (Hengl *et al*., 2017). Median effect size and quantile ranges (66% and 95%) are shown as black dots and intervals. The effects of cation exchange capacity were highly dependent on grid cell resolution and models, meaning effect sizes should be interpreted with caution (Fig. S7).

**Fig. 4:**
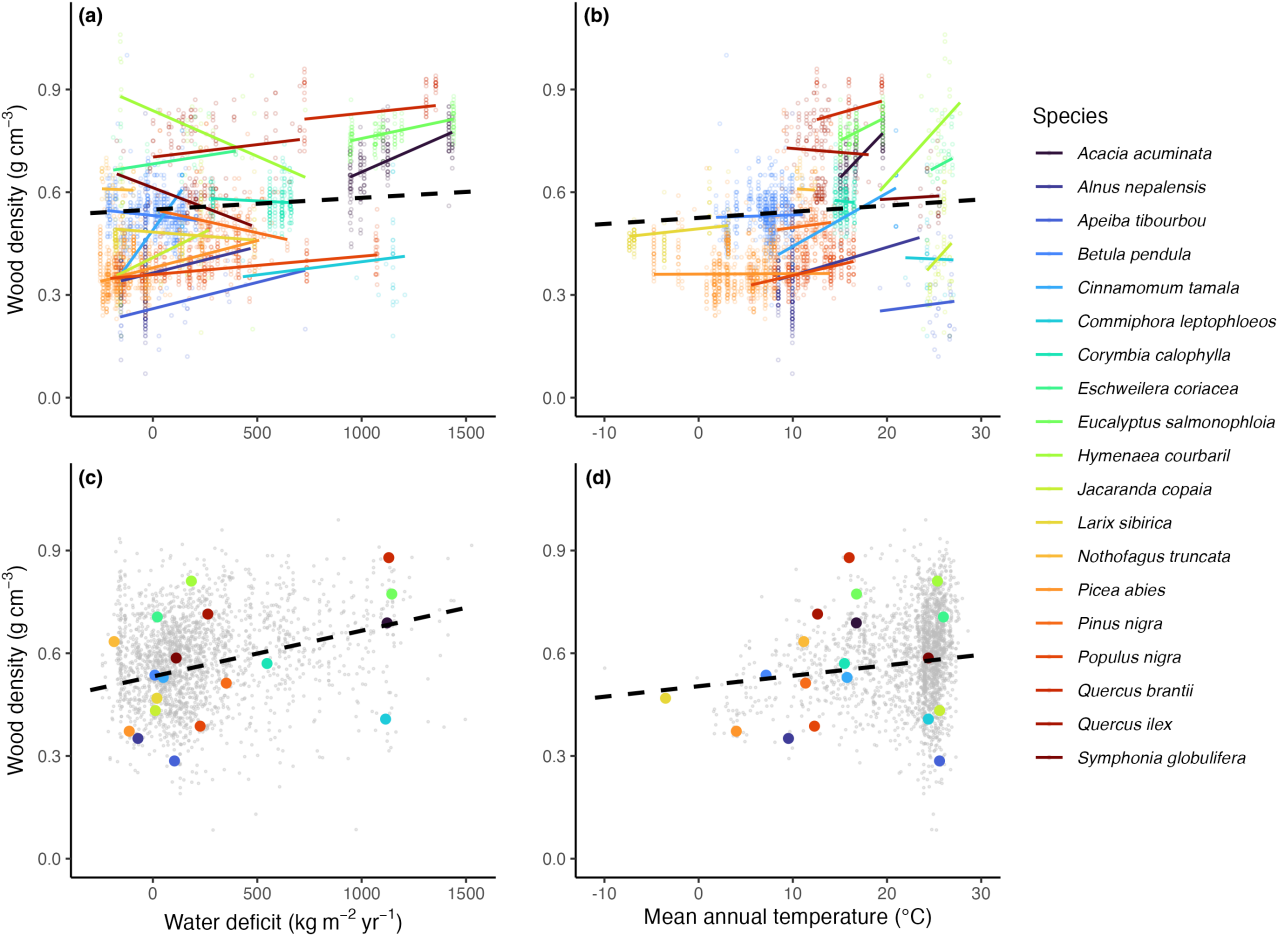
Effects of water deficit and temperature on wood density. Shown are the effects of climatic water deficit and mean annual temperature on wood density, as estimated from a hierarchical model (M16) and a large set of geolocated wood density records (*n*_species_ = 2160). Panels (a, b) show mean intraspecific effects (dashed black slopes) as well as species‐specific slopes and raw data for 19 species that cover a wide geographic and wood density range (in colour, *n* = 3224, > 20 samples per species). Panels (c, d) show the mean among‐species effect (dashed black slopes), species means for all 2160 species (small grey dots), with the 19 species' mean values highlighted (large dots). The model was fitted with standardized predictors, and results backtransformed to original scales for visualization. Enlarged versions of (a, b) with improved accessibility are provided as Supporting Information (Figs S15, S16).

Environmental models predicted wood density measurements well (*R* ^2^ = 0.71–0.73, RMSE = 0.062–0.069 g cm^−3^, models M16–M19) and better than a purely taxonomic model (*R* ^2^ = 0.63–0.64, RMSE = 0.072–0.079 g cm^−3^, M26), with small but consistent environmental effects on intraspecific wood density variation (Figs 3, S7–S10; Tables S10–S13). Wood density increased with temperature by 0.012 g cm^−3^ (standardized effect size) and with water deficit by 0.010 g cm^−3^; it decreased weakly with wind speed and soil pH (by −0.003 for both, Tables S10, S11). These effects were strongly correlated with interspecific effects (*r* = 0.83; Fig. S11), but were smaller by 70–80% (Figs 3, S7–S10; Tables S10–S13) and varied from one species to another (Fig. 4). For example, a typical intraspecific increase in wood density by 0.010 g cm^−3^ amounted to *c*. 25% of the respective interspecific effect (0.038 g cm^−3^), and species varied widely around this mean (SD = 0.038 g cm^−3^; Table S10).

Patterns were comparable when modelling tropical and extratropical species separately, but in the tropics, increases with water deficit (0.012 g cm^−3^ within and 0.059 g cm^−3^ among species) and decreases with pH (−0.014 within and −0.033 g cm^−3^ among species) were stronger. In a separate analysis for gymnosperms, there was a clear intraspecific decrease of −0.010 g cm^−3^ with soil pH and a strong intraspecific increase with water deficit (0.034 g cm^−3^; Table S14; Fig. S12). Since some species covered a narrow environmental gradient, effects were weaker when rescaling effect sizes to realized environmental ranges (Table S15; Figs S13, S14). For example, across a species' temperature range (95% interval), tropical wood density increased by an average of 0.012 g cm^−3^, and across a species' water deficit range by 0.032 g cm^−3^. Variation was much larger in some species, with increases from 0.64 to 0.78 g cm^−3^ for a water deficit range of 950 to 1430 kg m^−2^ yr^−1^ in *Acacia acuminata* Benth., but decreases from 0.65 to 0.50 g cm^−3^ between −170 and 470 kg m^−2^ yr^−1^ in *Symphonia globulifera* L.f. (Fig. 4a).

### 5. The effect of intraspecific variation on wood density estimation

Intraspecific variation strongly reduced the accuracy of wood density estimates in undersampled species. A single wood density measurement was a poor approximation of the species mean under cross‐validation (RMSE = 0.084 g cm^−3^); it was, in fact, comparable to a genus mean that did not involve any sampling of the target species (RMSE = 0.083 g cm^−3^; Table S16). However, the accuracy of species‐level wood density estimates improved quickly with better sampling. From the average of two measurements, species‐level wood density could already be predicted with RMSE = 0.056 g cm^−3^ (Table S16). Accuracy improved further when applying hierarchical models that combined individual measurements with taxonomic information from the remainder of the GWDD v.2 (RMSE = 0.038 g cm^−3^ for two measurements; Tables S16, S17).

**Fig. 5:**
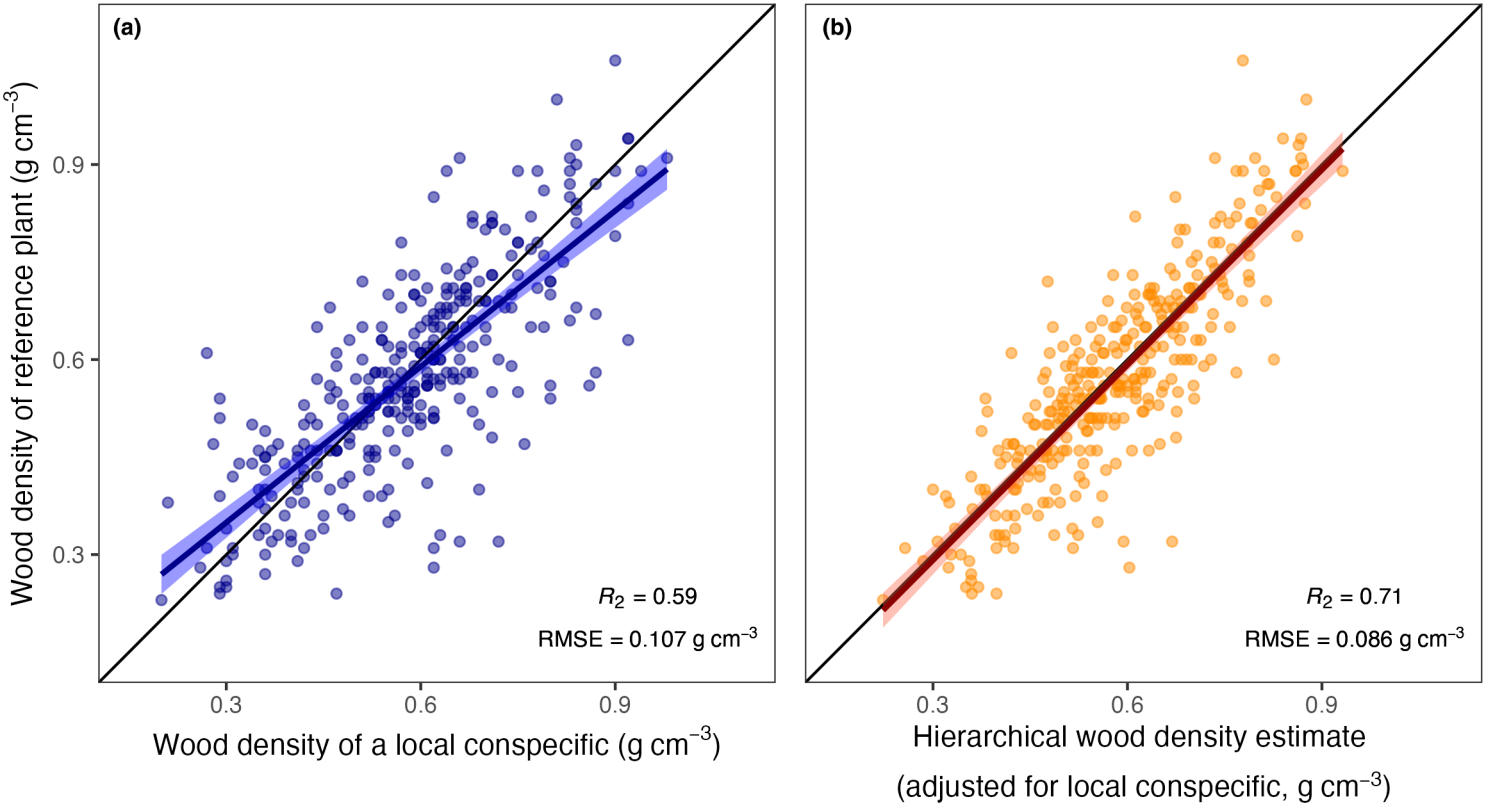
Prediction of an individual plant's wood density from a local conspecific. Shown are two approaches to estimate an individual plant's wood density when a measurement from a local conspecific is available: (a) using the conspecific's raw wood density measurement as an estimate and (b) using species or genus means adjusted by the local conspecific's value via hierarchical models (Model M27, Supporting Information Table S3). The black line is the 1 : 1 line, coloured lines and shaded areas are simple linear regressions and 95% confidence intervals. *R* ^2^ and root mean square errors (RMSEs) from linear regression are provided in the bottom right corner.

The wood density of individual plants was much harder to predict from conspecifics, with an RMSE of 0.107 g cm^−3^ and *R* ^2^ = 0.59 when estimated from a single local wood density measurement (Fig. 5a; Tables S16, S17). Errors were lower when pooling information with the GWDD v.2 via hierarchical modelling, but the improvements were moderate (RMSE = 0.086 g cm^−3^ or *c*. 20% of the raw estimate, and *R* ^2^ = 0.71, Fig. 5b). Errors were similar when three measures from local conspecifics were included, both as simple average (RMSE = 0.082 g cm^−3^) or based on a hierarchical model (RMSE = 0.083 g cm^−3^; Table S17).

## IV. Discussion

Wood density is a key trait in plant ecology, acting as an indicator of species' competitive abilities (Kunstler *et al*., 2016), demographic rates (Adler *et al*., 2014) and ecological strategies (King *et al*., 2006; Chave *et al*., 2009; Kraft *et al*., 2010). The importance of species‐level differences in wood density is well known (Phillips *et al*., 2019). However, intraspecific variation is commonly considered less relevant due to the large amounts of wood density variation explained by taxonomic and phylogenetic relationships (Chave *et al*., 2009), the strong genetic control over wood density (Cornelius, 1994; Zobel & Jett, 1995) and a limited contribution to community‐level variation in comparison with other plant traits (Siefert *et al*., 2015). Given that intraspecific variation in traits plays a crucial role for ecological processes (Bolnick *et al*., 2011; Des Roches *et al*., 2018) and influences biomass estimates (Nogueira *et al*., 2007; Momo *et al*., 2020), we here re‐examined this hypothesis at a global scale via the newly assembled Global Wood Density Database v.2, which, compared with the previous version (Zanne *et al*., 2009), included > 6× as many records, more than doubled the number of species, and added key information on sources of intraspecific variation.

### 1. Intraspecific variation in wood density matters

In the GWDD v.2, intraspecific variation in wood density (SD = 0.068 g cm^−3^) was substantial and structured according to both internal factors (within‐plant structure) and external factors (environment). Intraspecific variation accounted for up to 15% of global wood density variation and followed predictable patterns with environmental factors. Wood density increased with water deficit and more weakly decreased with soil pH. These results were consistent across models and datasets (Tables S10–S13) and confirmed expectations that plants with high wood densities should be favoured in extreme and nutrient‐poor environments (Muller‐Landau, 2004; Gourlet‐Fleury *et al*., 2011; Ibanez *et al*., 2017; Anderegg *et al*., 2021; Yang *et al*., 2023). There was also a general trend of increases in wood density with temperature, both in and out of the tropics, which may also reflect higher risks of drought‐induced embolisms in hotter environments, but it was not as clear as trends with water availability (Tables S10–S15). Crucially, environmental effects at intraspecific level were strongly correlated with interspecific effects (*r* = 0.83; Figs S11, S17) and were also consistent with a companion study that found that community‐means of wood density increased with temperature and aridity (Fischer *et al*., 2025). This consistency of environmental effects across scales was surprising, since previous studies found that variation in plant traits is often shaped by scale‐dependent physiological and ecological mechanisms (Anderegg *et al*., 2018; Wang *et al*., 2022; Zhou *et al*., 2022; Fajardo *et al*., 2024). Wood density, with its genetic limitations on variation, might thus be an exception, with similar physiological constraints operating across different levels of ecological organization.

We also found that previously hypothesized patterns of variation in wood density within individuals (Woodcock & Shier, 2002; MacFarlane, 2020) generalized to global scales. Across species and studies, wood density varied less in sapwood than in heartwood and less in branchwood than in trunkwood. Species with low‐density heartwood had denser sapwood and species with low‐density trunkwood had denser branchwood, and vice versa in both cases (Fig. 2a,b,d). An explanation for the heartwood‐sapwood trends may lie in distinct ecological strategies with changes in ontogeny (Wiemann & Williamson, 1988; Woodcock & Shier, 2002; Hietz *et al*., 2013), that is that some species tend to grow fast early in their life, investing little in dense wood, but slow down in later life. The opposite strategy – investing first in dense wood and then accelerating diameter growth later in life – has also been suggested, but appears less common (Osazuwa‐Peters *et al*., 2014). Trunk‐branchwood patterns have been explained through stronger functional constraints on branches than on trunks (MacFarlane, 2020; Momo *et al*., 2020), but it has also been argued that they may be explained by sapwood fractions (Gartner, 1995), since corewood formation at the tip of the stem occurs at the same time as the formation of outer wood at its base (cf summary in Wiemann & Williamson, 2013). Indeed, when we compared only sapwood samples, differences between branch and trunkwood largely disappeared (Fig. 2c). These results need to be qualified, however. While we found clear global patterns, our findings relied on coarse, binary distinctions between woody tissue types and thus glossed over methodological differences between studies, as well as finer biological details. Changes in wood properties from the pith to bark or from the base of trunk to branches often follow nonlinear patterns and vary strongly between individual plants (Schuldt *et al*., 2013; Osazuwa‐Peters *et al*., 2014; Terrasse *et al*., 2021), both of which remain to be studied in future research.

Our study also demonstrated that intraspecific variation in wood density has implications for applications, such as carbon stock assessments or functional ecology. It is common practice to use individual wood density measurements as estimates for species means (Réjou‐Méchain *et al*., 2017), but we found that individuals were generally so variable that a species mean was less accurately estimated from a single species measurement than from a genus mean. When applying hierarchical models, we found strong improvements in predictive accuracy for species means (from RMSEs g cm^−3^ of 0.084 down to 0.038 g cm^−3^ with two samples). However, increased sampling and hierarchical models did not help with predicting an individual plant's wood density. Even from three samples of local conspecifics, RMSEs did not decrease below 0.082 g cm^−3^, indicating that there was substantial biological variation among and within individuals that could not be explained by site‐specific environmental factors or phylogenetic relatedness. We note, however, that our study could not examine the local growth conditions and competitive neighbourhood of individuals, which likely play an important role in explaining intraspecific variation in wood density (Kunstler *et al*., 2016).

### 2. But intraspecific variation in wood density is also limited

Despite the importance of intraspecific variation in wood density, we still found that its extent was limited. First, it accounted for less of the total variance than taxonomic variation at species (17%), genus (30%) or family (30%) levels. Second, average environmental effects and wood density differences within individuals were generally small (usually *c*. 0.01 g cm^−3^ or less, exceptionally *c*. 0.02 g cm^−3^). They amounted to only 20–30% of species‐level effects and were outweighed both by methodological uncertainties, as may arise from differences in drying temperatures or wood coring (estimated at SD = 0.049 g cm^−3^ when counting only between‐study differences, Model M1, Tables 1, S4), and large among‐species variation in the direction and magnitude of intraspecific effects (Fig. 4). For example, across species, we found an average decrease of −0.023 g cm^−3^ from trunk to branchwood, but species varied in this relationship with SD = 0.075 (Model M10, Table S8). This result means that branchwood density was still higher than trunkwood density by > 0.015 g cm^−3^ in *c*. 30% of species. Similarly, despite an intraspecific increase of wood density with water deficit of 0.010 g cm^−3^, species varied in this effect with SD = 0.038 g cm^−3^ (Model M16, Table S10), meaning that in *c*. 30% of species wood density decreased with water deficit by a similarly sized 0.010 g cm^−3^. Even among well‐sampled species we found that wood density shifts could vary from as low as −0.15 g cm^−3^ to as high as +0.12 g cm^−3^ across a species' water deficit range (Fig. 4).

It is possible that these estimates of uncertainty and variation in effect sizes were inflated. The GWDD v.2 is a large global database, which unites data from a wide range of sources, increasing the risk of unbalanced sampling and confounding factors. For example, many measurements lacked information on geolocation and within‐plant location of samples (heartwood or sapwood, branch or trunk). Equally, it is often unclear how volume and mass were determined – directly in the field or after re‐immersion in water (Fearnside, 1997) – and at what temperatures wood samples were dried, which introduces additional variation into estimates (Williamson & Wiemann, 2010). We accounted for these differences by including the sampling source in models, fitting a range of models for each question and testing different GWDD v.2 subsampling strategies. While this revealed robust patterns at aggregate scale, interpretability of results for individual species remained limited. For example, if a species was measured by two studies in different locations and without clear documentation of methodology, it was not possible to conclusively attribute the sources of variation, and this may explain some of the uncertainty in effect sizes seen even among well‐sampled species (Fig. 4). Further issues include scale mismatches, for example between environmental predictors provided at 1 or 5 km resolution and wood density variation at finer scales (small‐scale elevation gradients, competition with neighbours, local growth conditions, such as light environment or waterlogging), as well as uncertainty in the predictors themselves. Branchwood definitions, for example, vary between studies, and global predictor layers come with considerable levels of uncertainty, which introduces both systematic errors and reduces effect sizes (Réjou‐Méchain *et al*., 2014). Most notably, the effect of cation exchange capacity on wood density varied strongly when assessed at 1 or 5 km scales (Tables S10, S11) and even changed qualitatively for a tropical subset of the GWDD v.2, for example from 0.027 to −0.021 g cm^−3^. It is likely that intraspecific variation in wood density could be better predicted if large aridity or temperature gradients were systematically sampled (Anderegg *et al*., 2021). However, even studies with systematic sampling designs across large gradients often find variable effects of the environment on intraspecific variation (Richardson *et al*., 2013; Rosas *et al*., 2019), and replications of our analyses with higher‐quality subsets of the GWDD v.2 did not generally affect results. For example, we still found the same small intraspecific increase with water deficit (0.008 g cm^−3^), the same large variation around the mean effect (SD = 0.039 g cm^−3^) and the same large effect of 0.045 g cm^−3^ among species (Model M18, Table S10).

Overall, this large variability in effect size and direction indicated that intraspecific wood density variation, even when following broad patterns, was difficult to predict. In practice, the inclusion of the location of tissues (branch vs trunk) into hierarchical models of wood density variation improved predictions at the species level from RMSE = 0.043 to 0.038 g cm^−3^ (assuming two wood density measurements, Table S16), but impacts on individual plant wood density estimates were minimal, with RMSE = 0.085 vs 0.084 g cm^−3^ (also assuming two wood density measurements, Table S17). These findings are consistent with several previous studies which found clear patterns for community‐ and species‐level wood densities (Swenson & Enquist, 2007; Chave *et al*., 2009; Kraft *et al*., 2010) where errors were smaller than total variation among taxa, but could not replicate these results at the intraspecific level, with patterns seemingly unpredictable (Richardson *et al*., 2013; Fajardo, 2018; Rosas *et al*., 2019; Umaña & Swenson, 2019). We also showed that environmental predictors had the potential to improve predictions, with *R* ^2^ = 0.71–0.73 compared with *R* ^2^ = 0.63–0.64 in a purely taxonomic model, but we did not build on this result, as we did not have consistent geographic information for the entire GWDD v.2. Future approaches might, for example, extend our approach by inferring species' climatic ranges and combine them with average intraspecific effects to arrive at more accurate wood density estimates for local‐scale analyses or unsampled species (Schrodt *et al*., 2015) and use Bayesian approaches that can handle missing data (Ogle *et al*., 2014). Based on reductions in RMSE in this study we estimated that predictive improvements would be in the range of 0.01–0.02 g cm^−3^, or < 5% of mean wood density.

### 3. When to account for intraspecific variation in wood density: a tale of two scales

Overall, our study showed that the decision of accounting for intraspecific variation in wood density depends on the scale of the research question. Measuring each individual's wood density and how it changes across its organs is paramount when studying plastic growth responses in individual plants. Intraspecific variation of up to 0.068 g cm^−3^ meant that two measurements of individuals from the same species could easily be separated by as much as ±0.19 g cm^−3^ (95% interval of the difference between two draws from a normal distribution with SD = 0.068 g cm^−3^). This variability should be large enough to overwhelm most species differences at a single site and led to large errors when predicting an individual's wood density, with *R* ^2^ as low as 0.59 and RMSEs as high as 0.108 g cm^−3^ in this study (Fig. 5). Intraspecific variation in wood density may thus dominate species‐level differences within communities or even across communities if these are dominated by only a few species that are close in average wood density values (cf patterns in Anderegg *et al*., 2021). Another case where intraspecific variation should be accounted for is the determination of individual tree biomass from terrestrial laser scanning (TLS). TLS‐derived 3D volumetric models can give precise volume estimates (Calders *et al*., 2015), which then must be combined with wood density to transfer volume to biomass. However, TLS estimates can be costly to construct, and their volume estimate precision only matters if wood density variation among individuals and along the hydraulic pathway is accounted for (Momo *et al*., 2020; Demol *et al*., 2021). Since we found errors of at least 0.08 g cm^−3^ when predicting wood density at the individual tree level (or between 10 and 20% of wood density, assuming most plants have densities between 0.40 and 0.80 g cm^−3^), studies likely cannot infer this variation but need to systematically sample multiple individuals with at least two samples per measurement location.

By contrast, if the aim is to assess average community structure and vegetation dynamics at large scales or across steep environmental gradients, it makes sense to prioritize taxonomic coverage (Phillips *et al*., 2019) over the exhaustive sampling of individuals from a single species. First, as we showed here, variation at species or higher taxonomic levels accounted for most variation in wood density (77%). Second, environmental effects at the intraspecific level aligned with interspecific effects (*r* = 0.83). Third, variation among and within individuals within sites is expected to average out at the community level. Therefore, as long as a wide range of communities is sampled, the omission of intraspecific effects should not introduce systematic bias. In some cases, such as wood density predictions via machine learning models (Yang *et al*., 2024), it may even make sense to ignore intraspecific information on purpose, as the risk of overfitting or mistaking small methodological differences for biological variation outweighs the benefits of small corrections of *c*. 0.01 g cm^−3^ or less. Similarly, intraspecific variation in wood density should play a minor role when applying precalibrated allometric models to estimate tree biomass, as its variance is dwarfed by other sources of uncertainty, such as allometric models and estimates of plant size and shape (Molto *et al*., 2013; Chave *et al*., 2014; Réjou‐Méchain *et al*., 2017; Kindermann *et al*., 2022).

### 4. Global traits databases as backbones for hierarchical models

A key takeaway of our study is that, no matter the level of analysis, wood density measurements should not be treated as monolithic true values. Rather, they are noisy trait estimates that can be refined by including prior information through shared evolutionary history or measurement locations (Ogle *et al*., 2014; Funk *et al*., 2017). Here, we applied simple hierarchical models based on taxonomic relationships and found that they vastly outperformed simple averaging procedures, particularly for undersampled species. A single wood density measurement was as poor an approximation of the species mean as a genus mean that did not involve any sampling of the target species (Table S16), but errors decreased substantially when combining both in a hierarchical model (Fig. 5; Tables S16, S17). Approaches could be refined by explicitly accounting for phylogenetic relationships, but this approach comes with its own challenges (Revell, 2010), and taxonomic hierarchies provide a good approximation (cf https://statmodeling.stat.columbia.edu/2016/02/14/hierarchical‐models‐for‐phylogeny‐heres‐what‐everyones‐talking‐about/, last accessed on 9 September 2024, also Ogle *et al*., 2014).

Overall, our findings suggest that there is great value in open‐access trait databases, such as the GWDD v.2, as they synthesize knowledge across a range of disciplines and help correct noisy local estimates. They also provide insights on ecological strategies, variation across biogeographic realms, and, with careful curation and documentation (Augustine *et al*., 2024), allow us to explore intraspecific variation. At the time of writing, v.1 of the GWDD has been downloaded almost 20 000 times. Many of the applications of this database have been in assessing forest carbon storage, in connection with REDD+ projects or carbon credit accounting programmes. As this sector is coming under closer scrutiny, reducing uncertainty in carbon estimates is a timely ambition, and the GWDD v.2 will be an important contribution. The findings in this study provide more robust wood density estimates for a much larger range of species and guidance on the importance of intraspecific variation and how to account for it in ecological studies. Furthermore, our results can be a foundation for theories about the evolution of carbon investments in plants (Castorena *et al*., 2022) and how to parameterize the underlying processes in global dynamic vegetation models. In the future, we hope that the openly available and thoroughly documented GWDD v.2 will encourage the documentation and sharing of more wood density datasets and the construction of similar databases for other traits.

## Competing interests

None declared.

## Author contributions

FJF, JC and AZ conceived of the project. FJF led the assembly and processing of the database, the analysis for the manuscript and the writing of a first draft of the manuscript, with assistance from JC, AZ, TJ, AF, AF, RAFL and GV. HB, WH, TDM, DW, AMA, EA‐D, LFA, DMGA, FA, JFB, AB, PB, VB, JB, VB, JJC, LC, RCG, JQC, ECF, BC, GC, WC, JAD, AKD, MD, DD, DPE, RE, DF, PF, OF, NF, JG, RCG, DG, LFH‐D, VH, PH, JH, TI, JI, SJ, RMK, TK, LK, SK, MK, YK, PL, JL, ALAL, RML, AL, RL, CM‐N, LFSM, ARM, AMM, JKM, RBM, AJN, BWN, MN, EMN, AO, RO, MO, YO, KP, DP, PR, OR, TR, JR, SR, EGR, ORR, SGR, VR, JAR, RS‐G, NSS, BS, LS, AS, TS, PRS, SS, MTS, BT, DYPT, JMDT, BV, AW, JW, SJW and KZ contributed substantially through data collection, data assembly and revisions of the manuscript.

## Disclaimer

The New Phytologist Foundation remains neutral with regard to jurisdictional claims in maps and in any institutional affiliations.

## Supporting information


**Fig. S1** Sample posterior predictive checks.
**Fig. S2** Variance components estimated from a Bayesian hierarchical model.
**Fig. S3** Intraspecific variation in selected species from six plant families.
**Fig. S4** Consistency of the extent of intraspecific variation across taxa.
**Fig. S5** Wood density variation between trunkwood and branchwood, high quality regressions.
**Fig. S6** Wood density variation in trunkwood and in branchwood in selected species.
**Fig. S7** Environmental and edaphic effects on wood density.
**Fig. S8** Environmental and edaphic effects on wood density, high‐quality subset.
**Fig. S9** Environmental and edaphic effects on wood density, within tropics.
**Fig. S10** Environmental and edaphic effects on wood density, outside of tropics.
**Fig. S11** Correlation between within‐species effects and between‐species effects.
**Fig. S12** Environmental and edaphic effects on wood density in gymnosperms.
**Fig. S13** Temperature effects on within‐species wood density variation, examples.
**Fig. S14** Water deficit effects on within‐species wood density variation, examples.
**Fig. S15** Intraspecific effects of climatic water deficit on wood density.
**Fig. S16** Intraspecific effects of mean annual temperature on wood density.
**Fig. S17** Correlation between environmental and edaphic predictors.
**Methods S1** Updating the first GWDD.
**Methods S2** Literature references for wood density values.
**Methods S3** Converting air‐ and oven‐dry density to basic wood density.
**Methods S4** Details on Bayesian modelling of wood density variation.
**Methods S5** Overview over R packages.
**Table S1** Documentation of GWDD v.2 columns.
**Table S2** Removed taxa in GWDD v.2.
**Table S3** Overview over models and data subsets.
**Table S4** Variance components of wood density across the taxonomic hierarchy (brms).
**Table S5** Variance components of wood density across the taxonomic hierarchy (lme4).
**Table S6** Variance components of wood density within species (brms).
**Table S7** Variance components of wood density within species (lme4).
**Table S8** Within‐plant effects on wood density variation (brms + lme4).
**Table S9** Within‐plant convergence in wood density (lmodel2).
**Table S10** Environmental predictors of wood density variation (brms).
**Table S11** Environmental predictors of wood density variation (lme4).
**Table S12** Environmental predictors of wood density variation, in and outside of tropics (brms).
**Table S13** Environmental predictors of wood density variation, in and outside of tropics (lme4).
**Table S14** Environmental predictors of wood density variation in gymnosperms (brms + lme4).
**Table S15** Rescaled environmental effect sizes, in and outside of tropics (brms).
**Table S16** Quality of wood density predictions at the species level.
**Table S17** Quality of wood density predictions at the individual plant level.
Please note: Wiley is not responsible for the content or functionality of any Supporting Information supplied by the authors. Any queries (other than missing material) should be directed to the *New Phytologist* Central Office.

## Data Availability

The Global Wood Density Database v.2 and derived wood density estimates at species and genus level are openly available on Zenodo (doi: 10.5281/zenodo.16919509). All data and code underpinning the results of this article are publicly archived in a separate Zenodo archive (doi: 10.5281/zenodo.16928342).
